# Person-centered and measured life’s simple 7 cardiovascular health concordance and association with incident cardiovascular disease

**DOI:** 10.1038/s41598-023-32219-x

**Published:** 2023-03-31

**Authors:** Omar Deraz, Thomas Van Sloten, Rachel Climie, Charlotte Debras, Léopold K. Fezeu, Mélanie Deschasaux-Tanguy, Xavier Jouven, Emmanuelle Kesse-Guyot, Pilar Galan, Serge Hercberg, Mathilde Touvier, Jean-Philippe Empana

**Affiliations:** 1 Université Paris Cité, UMR-S970, Paris Cardiovascular Research Center, INSERM, Integrative Epidemiology of Cardiovascular Disease (Team 4), Paris, France; 2 Department of Vascular Medicine, University Medical Center Utrecht, Utrecht, the Netherlands; 3 Menzies Institute for Medical Research, University of Tasmania, Hobert, Australia; 4 Baker Heart and Diabetes Institute, Melbourne, Australia; 5 Nutritional Epidemiology Research Team (EREN), Sorbonne Paris Nord University, Inserm U1153, Inrae U1125, Cnam, Epidemiology and Statistics Research Center – University of Paris (CRESS), Bobigny, France

**Keywords:** Cardiovascular diseases, Cardiology, Epidemiology, Biomarkers

## Abstract

Person-centered cardiovascular health (CVH) may facilitate cardiovascular disease primordial prevention in low resources settings. The study aims to assess the validity of person-centered CVH compared to gold standard measured CVH by examining the concordance between person-centered vs. measured CVH together with their respective association with incident cardiovascular disease events (CVD). Life’s Simple 7 (LS7) CVH metrics, including non-smoking, Body Mass Index, diet, physical activity, blood glycemia, blood pressure, and blood cholesterol were collected from 19,473 adults participating in the e-cohort NutriNet-Santé study from 2011 to 2014 and were followed until September 2020. Clinical examinations and blood analyses defined the measured biological metrics, while diagnoses, medication, or treatment for type 2 diabetes, hypertension, and hypercholesterolemia defined person-centered biological metrics. Declared behavioral metrics were common for both measured and person-centered CVH. The study included 18,714 CVD-free participants (mean age 51 years, 73% women), among whom 16.52% and 38.75% had 5–7 ideal LS7 metrics according to measured and person-centered CVH, respectively. Weighted concordance of person-centered and measured CVH was 0.87 [0.86; 0.88]. Over median follow-up of 8.05 years, 749 CVD events occurred. There was a 7% (HR 0.93 [0.88; 0.99]) and 13% (HR 0.87 [0.83; 0.92]) risk reduction of CVD risk by additional measured and person-centered ideal metrics, respectively. In conclusion, person-centered CVH may represent a reliable alternative to measured CVH.

## Introduction

Cardiovascular diseases (CVD) are associated with the highest disease burden globally, with a 2019 prevalence of 523 million, nearly double the figure observed in 1990. During the same period, CVD mortality increased from 12.1 million 95% UI [11.4; 12.6] to 18.6 million 95% UI [17.1; 19.7]. The contribution of CVD to Disability Adjusted Life Years (DALYs) doubled in 2019 compared to 1990^1^. While primary prevention has been shown to explain more than half of the decline in CVD mortality in industrialized countries^2^, the figures above underline the need to do more, particularly in low and middle-income countries (LMIC) that are witnessing an epidemiological transition^3^. In addition to controlling risk factors once they are in place (primary prevention), preventing their onset in the first place might be an additional complementary preventative strategy, the so-called primordial prevention.

Primordial prevention is increasingly recognized as a key strategy for early CVD population prevention^4^. To this end, the American Heart Association defined Life’s Simple 7 (LS7) cardiovascular health (CVH) score, which includes four behavioral metrics (non-smoking, and ideal levels of body mass index, physical activity, and diet), and three biological metrics (untreated ideal levels of fasting plasma glucose, blood pressure, and total cholesterol), as a tool to monitor the level of cardiovascular health at the population level^5^. The clinical relevance of CVH has been established in various populations, whereby a higher CVH level is associated with lower CVD incidence^6^. However, the prevalence of ideal CVH, i.e. 5 or 6 to 7 ideal LS7 metrics, remains low, below 15% in the adult population^6,7^. Widespread use of cardiovascular health score is hindered by the prerequisite of clinical examinations and blood analyses. Moreover, blood biomarker measurements are particularly challenging to acquire in LMIC. From a global health perspective, given the high burden of CVD, the high prevalence of CVD risk factors, as well as socioeconomic disadvantages in LMIC^1,8^, one avenue to improve ideal CVH figures could be to adopt a person-centered CVH promotion model. Person-centered care approaches whereby individuals are informed and actively participate in their care are rapidly developing^9^. This approach could be extended to primordial prevention in which individuals have agency over their prevention and are empowered to adopt health-promoting behaviors. Nonetheless, the validity of person-centered CVH compared to the gold standard measured CVH and direct comparisons of their respective associations with incident CVD is currently unidentified. Therefore, the main study objective is to assess the validity of person-centered CVH compared to gold standard measured CVH. To address this question, (1) the concordance between person-centered vs. measured LS7 metrics was investigated; and (2) to provide clinical relevance to the findings, a qualitative comparison was conducted for the associations of person-centered vs. measured LS7 metrics with incident CVD.

## Methods

The NutriNet-Santé Study (https://clinicaltrials.gov/, Registration Number: NCT03335644) is an ongoing web-based e-cohort launched in 2009 that examines the relationship between nutrition and health and determinants of dietary patterns and nutritional status^10^. The study recruited 171,000 participants aged ≥ 18 years from all over France with internet access. Between 2011 and 2014, blood measurements and physical examinations were recorded in a subsample of 19,473 participants, who represent the target population for the main analysis. The study was approved by the Institutional Review Board of the French Institute for Health and Medical Research and the National Commission on Informatics and Liberty, CNIL (n°908,450/ n°909,216). The participants consented after being informed about the study objectives and details. All research procedures were conducted in accordance with the declaration of Helsinki and relevant guidelines and regulations.

### Data acquisition

The participants completed annual validated questionnaires on the NutriNet-Santé online platform (https://etude-nutrinet-sante.fr/). The questionnaires included sociodemographic and lifestyle questionnaires, comprising data on smoking of cigarettes, cigars, and pipes, an International Physical Activity Questionnaire (IPAQ)^11^, an anthropometric questionnaire for weight and height^12,13^, and a series of three nonconsecutive multi-seasonal web-based 24-h dietary records on two weekdays and one weekend day^14–16^. Recurrent 3-month questionnaires collected information on medical treatment and disease conditions.

During a clinical examination, height was measured by a stadiometer to the nearest 0.5 cm, and weight was measured by a calibrated scale to the nearest 0.1 kg (body composition analyzer BC-418MA, TANITA, Tokyo, Japan). Blood pressure was measured 3 times at 1-min intervals in a seated position after 5 min of rest using an automatic sphygmomanometer (Omron HEM-7015IT^®^; OMRON). The average of the recorded values was used in the present study. The participants were requested online to visit a participating Local Sample Collection Centers (LSCC) equipped for blood sample draws. Total serum cholesterol was measured by enzymatic cholesterol oxidase (Architect c8000^®^, Abbott). Fasting plasma glucose (FPG) was measured by hexokinase/G-6-PDH (Architect c8000^®^, Abbott).

### CVH metrics

#### Definitions

Table 1 lists the definitions of person-centered and measured CVH metrics. Ideal levels for the behavioral metrics correspond to BMI < 25 kg/m^2^, never smoker and quitter > 12 months, ≥ 75 min/week of vigorous physical activity, ≥ 150 min/week of moderate physical activity, or a combination of the two, and ≥ 4 optimal diet items (the equivalent of 5 servings per day of fruits and vegetables or 400 gm/day (according to the national French nutrition and health program), the equivalent of fish consumption ≥ 2 times/week or average consumption of ≥ 100 gm/day, average sodium consumption < 1.5 gm/day, average fiber intake ≥ 25 gm/day, average intake of sugary drinks ≤ 152 ml/day). The ideal levels for the measured biological metrics correspond to untreated fasting blood glucose < 100 mg/dl, untreated blood pressure (BP) < 120/80 mmHg, and untreated total serum cholesterol < 200 mg/dl. The ideal level for the person-centered biological metrics corresponds to the absence of declared medication, treatment, or diagnosis for type 2 diabetes (T2D), hypertension, or hypercholesterolemia. The main exposure is the number of person-centered and measured ideal metrics (range 0–7). The number of person-centered and measured ideal metrics was also categorized as 0–2, 3–4, 5–7 ideal metrics to denote low, intermediate, and high CVH levels^17^.

**Table 1:** Definitions of measured and person-centred cardiovascular health Life’s Simple 7 metrics.

	Ideal level	Intermediate level	Poor level
Behavioural LS7 metrics common to measured and person-centred CVH			
Non-smoking	Never or former smoker ≥ 12 months	Former < 12 months	Current smokers
Body mass index	< 25 kg/m^2^	25–29.9 kg/m^2^	≥ 30 kg/m^2^
Physical activity	MET ≥ 75 min/week of vigorous activity, MET ≥ 150 min/week of moderate activity, or a combination of the two	1–74 MET/week vigorous activity, 1–149 MET/week moderate activity or a combination of both	None
Healthy diet	≥ 4 optimal diet items out of the following 5 items: the equivalent of 5 servings per day of fruits and vegetables or 400 gm/day, ≥ 2 times/week fish consumption or average consumption of ≥ 100 gm/day, average sodium consumption < 1.5 gm/day, average fiber intake ≥ 25 gm/day, average intake of sugary drinks ≤ 152 ml/day	2–3 optimal diet items out of the following 5 items: the equivalent of 5 servings per day of fruits and vegetables or 400 gm/day, ≥ 2 times/week fish consumption or average consumption of ≥ 100 gm/day, average sodium consumption < 1.5 gm/day, average fiber intake ≥ 25 gm/day, average intake of sugary drinks ≤ 152 ml/day	≤ 1 optimal diet items out of the following 5 items: the equivalent of 5 servings per day of fruits and vegetables or 400 gm/day, ≥ 2 times/week fish consumption or average consumption of ≥ 100 gm/day, average sodium consumption < 1.5 gm/day, average fiber intake ≥ 25 gm/day, average intake of sugary drinks ≤ 152 ml/day

Measured biological LS7 metrics			
	Ideal level	Intermediate level	Poor level
Fasting plasma glucose	< 100 mg/dL, untreated	100–126 mg/dL or < 100 mg/dL treated to goal	> 126 mg/dL
Blood pressure	SBP < 120 and DBP < 80 mmHg, untreated	SBP 120–139 and DBP 80–89 mmHg or SBP < 120 and DBP < 80 mmHg and treated to goal	SBP ≥ 140 and DBP ≥ 90 mmHg
Total serum cholesterol	< 200 mg/dL, untreated	200–240 mg/dL or < 200 mg/dL treated to goal	> 240 mg/dL

Person-centered biological LS7 metrics			
	Ideal level	Non-ideal level	
Type 2 diabetes	No diagnosis, medication, or treatment	Reported diagnosis, medication, or treatment	
Hypertension	No diagnosis, medication, or treatment	Reported diagnosis, medication, or treatment	
Hypercholesterolemia	No diagnosis, medication, or treatment	Reported diagnosis, medication, or treatment	

*MET* metabolic equivalents min/week, *SBP* systolic blood pressure, *DBP* diastolic blood pressure.

### Data sources

The completed questionnaires were used to define the person-centered behavioral metrics and the biological metrics, while the data collected during the physical examination (blood pressure) and blood tests (total serum cholesterol and fasting plasma glucose) were employed to define the measured biological metrics. Person-centered weight and height were shown to be adequately concordant with their measured counterparts in the Nutrinet-Santé Study, with an interclass correlation ICC 0.97 of measured and person-centered BMI^12,13^.

### Alignment of health questionnaires and clinical appointment

The following approach was used to align the dates of health questionnaires (person-centered biological and behavioral metrics) with the dates of clinical appointment (measured biological metrics): for participants whose clinical appointment date was < 2 years from their recruitment date, their first questionnaire-based CVH metrics were used to define person-centered biological and behavioral metrics; and for those whose clinical appointment date was ≥ 2 years from their recruitment date, their follow-up questionnaire-based CVH metrics the closest to the clinical appointment date, which ensured a lag time less than two years were selected (time range: 0.55 to 63.52 months, Supplementary Fig. S1). In any case, the date of clinical appointment was used as the starting point for CVD events follow-up (see “Statistical methods”).

### Covariates

The Educational attainment categories were (1) less than high school, (2) less than 2 years after high school), and (3) more than or equal to 2 years after high school. The occupational categories were (1) liberal profession: farmer, craftsperson, merchant, company director, specialized personnel, (2) intellectual profession, (3) intermediary profession, and (4) worker/employee. Alcohol use was calculated as daily average ethanol consumption (gm/day). CVD family history was defined by declared first-degree relative with CVD history.

### CVD ascertainment

The primary outcome was fatal and non-fatal CVD events as of September 2020. The participants reported health events through a yearly health questionnaire, a check-up questionnaire every 3 months, or at any time through a dedicated platform on the study’s website. Participants’ families or doctors were contacted in case of no response on the study website for more than 1 year. A committee of physicians reviewed the medical data to validate major health event diagnoses, hospital admissions, and records, i.e. radiological reports and electrocardiograms. If necessary, the study doctors contacted the participants’ doctors or medical facilities, i.e. clinics, hospitals, or laboratories, to collect additional information. The data from the population-based cohort was linked to the medico-administrative databases of national health insurance (SNIIRAM); thus, limiting potential bias induced by unreported CVD. Fatal or non-fatal CVD cases were defined by the *International Classification of Diseases ICD-10-CM*, Tenth Revision. Hemorrhagic/ischemic stroke (ICD-10-I64), transient ischemic attack (ICD-10-G45.8 and ICD-10-G45.9), angina pectoris (ICD-10- I20.9), acute coronary syndrome (ICD-10- I24.9 and ICD-10-I21.4), myocardial infarction (ICD-10-I21), sudden cardiac death (ICD-10-I46.1, ICD-10-I46.9), coronary angioplasty (ICD-10-Z95.5), were included. For deceased participants, the causes of death were ascertained by linkage to the national registry of the causes of death, Center for Epidemiology of Causes of Death (CépiDC).

### Statistical methods

The percentage agreement between low, intermediate, and high levels of the measured and person-centered CVH; percentage agreements between measured poor, intermediate, and ideal biological metrics vs. ideal/non-ideal person-centered biological metrics were estimated. Such analysis was not conducted for the behavioral LS7 metrics since they are common to person-centered and measured CVH. Weighted Gwet AC2/Brennan and Prediger coefficients assessed the inter-rater reliability (IRR) of the person-centered and measured number of ideal LS7 metrics (range 0–7)^18,19^, and they were quadratically weighed to allocate more weight to farther misclassifications of person-centered CVH according to the measured CVH level. Their value ranges from 0 to 1; the closer to 1, the more substantial the concordance. Sensitivity (Se), specificity (Sp), negative and positive predictive value test concordance statistics (NPV and PPV), and diagnostic accuracy (i.e. the proportion of correctly classified participants) were calculated using the measured CVH as a gold standard. These statistics were computed for the global CVH and individual biological metrics.

For survival analysis, person-time started at the date of the clinical appointment, and the event date was the first non-fatal CVD event, fatal CVD event, or mortality. Event-free participants’ follow-up was censored at their last answered questionnaire. Kaplan–Meier curves were used to plot the cumulative incidence of CVD using time-in-study, according to the low (0–2), intermediate (3–4), and high (5–7) measured and person-centered CVH levels. Survival curves were compared across CVH levels using the log-rank trend test. The hazard ratios (HR) and their 95% confidence intervals (CIs) for incident CVD were estimated per one point increase in the number of ideal LS7 metrics and for intermediate or high vs. low measured and person-centered CVH. Cox proportional hazard models using age as the time scale were stratified by birth-year (5-year intervals) and were adjusted for prespecified covariates including baseline sex, cohabitation status, educational attainment, occupational category, alcohol use, and CVD family history. The proportionality assumption was assessed using Schoenfeld’s residuals plot/test and negative log (log survival) plots. The linearity assumption between ideal metrics and incident CVD was tested via martingale residual analysis. Multiple imputations by chained equations were used to handle missing LS7 metrics (n = 2417) and missing covariates (n = 585), using linear or logistic regression models adjusted for demographic and socioeconomic variables. Fifty imputed datasets were produced, and the estimation method was Rubin’s combination rule.

### Additional analyses

The robustness of the study results was evaluated by the following additional analyses. To assess the potential influence of latent CVD, the analyses were conducted by excluding participants who developed CVD during the first year of follow-up. To further explore to which extent the difference in definition between person-centered vs. measured biological metrics impacted the HR estimate, the hazard ratio estimates were computed exclusively for the biological LS7 metrics (i.e. per number of ideal metrics, ranging 0–3). Complete case analysis was conducted to evaluate the potential impact of missing values on the regression estimates. The competing risk of non-CVD mortality was estimated using the Fine and Gray method^20^. Given prior evidence relying measured CVH with non-CVD mortality^7^, the association of person-centered CVH with non-CVD-mortality was further evaluated. Population attributable preventive fraction was estimated for the hypothetical scenario in which all the population would have attained current high-level CVH. Lastly, to further evaluate the relevance of person-centered CVH, the association between change in person-centered CVH over 5 years and subsequent risk of CVD was assessed in the full sample recruited since 2009. The data retrieval, management, and statistical analyses were carried out using SAS Enterprise Guide^®^ 7.1, Oracle^®^ 12.2.0., and Stata^®^ 17.0 (StataCorp, Tex).

## Results

### Main characteristics

**Figure 1:**
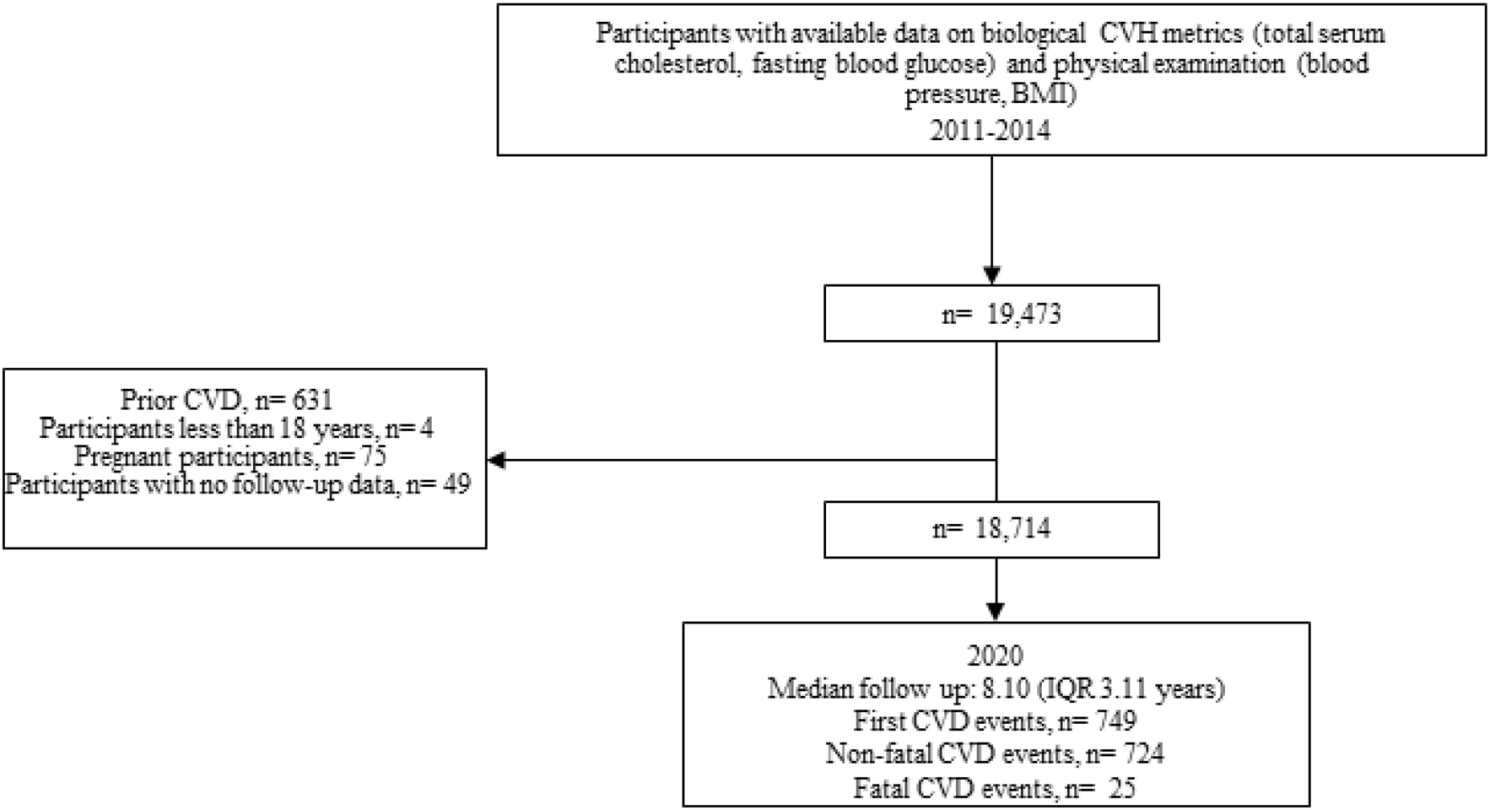
Study sample flowchart.

The study population included 18,714 adult CVD-free participants (Fig. 1), average age 51 years, 73% women. Table 2 summarizes the sample characteristics according to the levels of measured and person-centered CVH. In both measured and person-centered CVH, people with higher CVH were more frequently younger (43.58, 48.60 years), women (77.59%, 75.63%), of higher educational attainment (44.72%, 38.94%), drinking less alcohol (6.90 gm/day, 5.56 gm/day), and less frequently having CVD family history (26.98%, 34.29%). The percentages of ideal person-centered and measured LS7 metrics are reported in Supplementary Table S1.

**Table 2:** Sample characteristics according to person-centered and measured CVH level.

	Person-centered CVH			P-trend	Measured CVH			P-trend	Overall
	Low	Intermediate	High		Low	Intermediate	High		
	1928 10.30%	9534 50.95%	7252 38.75%		5385 28.78%	10,237 54.70%	3092 16.52%		18,714
Mean age (SD) years	59.82 (8.74)	51.48 (13.11)	48.60 (14.08)	< 0.05	56.73 (11.57)	50.65 (8.25)	43.58 (5.56)	< 0.05	51.22 (13.51)
Women	55.96%	73.62%	75.63%	< 0.05	62.47%	76.48%	77.59%	< 0.05	72.58%
Educational attainment									
< high school	25.41%	16.87%	13.40%		22.57%	15.22%	9.63%		16.40%
< 2 years after high school	49.01%	49.47%	47.66%		49.35%	49.32%	45.65%		48.72%
≥ 2 years after high school	25.57%	33.67%	38.94%	< 0.05	28.08%	35.46%	44.72%	< 0.05	34.88%
Occupational category									
Liberal profession	4.67%	4.70%	5.41%		4.65%	4.95%	5.80%		4.97%
Intermediary profession	29.77%	29.88%	29.33%		29.96%	29.94%	28.01%		29.66%
Intellectual profession	42.74%	40.04%	41.49%		40.66%	40.59%	41.37%		40.88%
Worker/employee	22.82%	25.38%	23.77%	0.02	24.73%	24.47%	24.79%	0.20	24.50%
Cohabitant	79.05%	79.19%	79.48%	0.90	79.77%	79.28%	78.46%	0.40	79.29%
Mean alcohol, gm/day	12.02	9.51	6.90	< 0.05	11.57	8.25	5.56	< 0.05	8.76
SD	14.82	12.14	9.35		14.25	10.55	8.16		11.59
CVD family history	58.35%	40.60%	34.29%	< 0.05	49.16%	39.11%	26.98%	< 0.05	39.99%

Values are percentages for categorical variables and mean (standard deviation) for numerical ones.

Low, intermediate, and high CVH correspond to 0–2, 3–4, and 5–7 ideal LS7 metrics, respectively.

Trend tests include Jonckheere trend test for numerical variables and the Chi-square trend test for categorical variables.

Educational attainment categories were (1) low (less than high school), (2) intermediate (less than 2 years after high school), and (3) high (more than or equal to 2 years after high school).

Occupational categories were (1) liberal professions (farmer, craftsperson, merchant, company director, specialized personnel, (2) intellectual professions, (3) intermediary professions, and (4) worker/ employee.

### Concordance between measured and person-centered CVH

As reported in Table 3, the concordance between low, intermediate, and high person-centered vs. measured CVH was high, with Gwet AC2 estimate of 0.92, 95% CI [0.91; 0.93], and Brennan and Prediger estimate of 0.87, 95% CI [0.86; 0.88]. The sensitivity and specificity of person-centered CVH were 96.94% and 72.83%, and the PPV and NPV were 41.52% and 99.17%, respectively; the diagnostic accuracy was 76.83% When examining the biological metrics separately (Table 4), the sensitivity ranged from 93.44% (hypertension) to 99.87% (T2D), and the specificity ranged from 15.37% (T2D) to 39.89% (hypertension), the PPV from 37.15% (hypercholesterolemia) to 88.14% (T2D) and the NPV from 79.60% (hypertension) to 95.95% (hypercholesterolemia) respectively. The diagnostic accuracy was 88.29%, 72.51%, and 46.86% for person-centered T2D, hypertension, and hypercholesterolemia, respectively.

**Table 3:** Validity of person-centered and measured ideal cardiovascular health (CVH).

Person-centered CVH levels	Measured CVH levels		
	Low	Intermediate	High
Low	1717 31.95%	211 2.06%	0 0.00%
Intermediate	3548 66.02%	5891 57.56%	95 3.06%
High	109 2.03%	4201 40.12%	3011 96.94%
Sensitivity	96.94% [96.28; 97.49]		
Specificity	72.83% [72.12; 73.52]		
PPV	41.52% [40.39; 42.66]		
NPV	99.17% [98.99; 99.32]		
Diagnostic accuracy	76.83% [76.22, 77.43]		
Gwet's AC2	0.92 [0.91; 0.93]		
Brennan and Prediger	0.87 [0.86; 0.88]		

*CVH* cardiovascular health, *LS7* life’s simple 7, *PPV* positive predictive value, *NPV* negative predictive value.

Low, intermediate, and high CVH correspond to 0–2, 3–4, and 5–7 ideal LS7 metrics, respectively.

Sensitivity, Specificity, PPV, and NPV Statistics were computed for ideal and non-ideal categories of person-centered and measured CVH.

Diagnostic accuracy was calculated using the proportion of a true ideal and true non-ideal measured classification according to the person-centered CVH among all the participants.

Weighted Gwet's AC2/ Brennan and Prediger statistics evaluated the concordance between the person-centered and measured number of ideal LS7 metrics.

**Table 4:** Validity of person-centered and measured biological Life’s Simple 7 cardiovascular health metrics.

Person-centered type 2 diabetes	Measured fasting plasma glucose level			
		Poor	Intermediate	Ideal
Non-ideal﻿		133	261	21
		32.05%	62.89%	5.06%
Ideal		90	2080	16,129
		0.49%	11.37%	88.14%
Sensitivity	99.87% [99.80; 99.91]			
Specificity	15.37% [14.02; 16.81]			
NPV	94.94% [92.39; 96.67]			
PPV	88.14% [87.66; 88.60]			
Diagnostic accuracy	88.29% [87.82; 88.74]			
	Measured blood pressure			
Person-centered hypertension				
		Poor	Intermediate	Ideal
Non-ideal		471	2447	748
		12.85%	66.75%	20.40%
﻿Ideal		906	3491	10,651
		6.02%	23.20%	70.78%
Sensitivity	93.44% [92.97; 93.88]			
Specificity	39.89% [38.77;41.02]			
NPV	79.60% [78.26; 80.87]			
PPV	70.78% [70.05; 71.50]			
Diagnostic accuracy	72.51% [71.86; 80.87]			
	Measured total serum cholesterol			
Person-centered hypercholesterolemia				
		Poor	Intermediate	Ideal
﻿Non-ideal		1109	1856	125
		35.89%	60.06%	4.05%
﻿Ideal		3472	6348	5804
		22.22%	40.63%	37.15%
Sensitivity	97.89% [97.49; 98.23]			
Specificity	23.19% [22.47; 23.93]			
NPV	95.95% [95.20; 96.59]			
PPV	37.15% [36.39; 37.91]			
Diagnostic accuracy	46.86% [46.14; 47.57]			

*PPV* positive predictive value, *NPV* negative predictive value.

Ideal level of measured fasting plasma glucose, blood pressure, and total serum cholesterol correspond to untreated fasting plasma glucose < 100 mg/dL, untreated systolic and diastolic blood pressure < 120/80 mmHg, and untreated total serum cholesterol < 200 mg/dL, respectively.

Corresponding definitions for person-centered LS7 metrics are the absence of medication, treatment, or diagnosis for type 2 diabetes, hypertension, or hypercholesterolemia. Sensitivity, Specificity, NPV, PPV Statistics were computed for ideal and non-ideal levels of measured and person-centered LS7 metrics.

### Association between measured and person-centered CVH metrics with incident CVD

**Figure 2:**
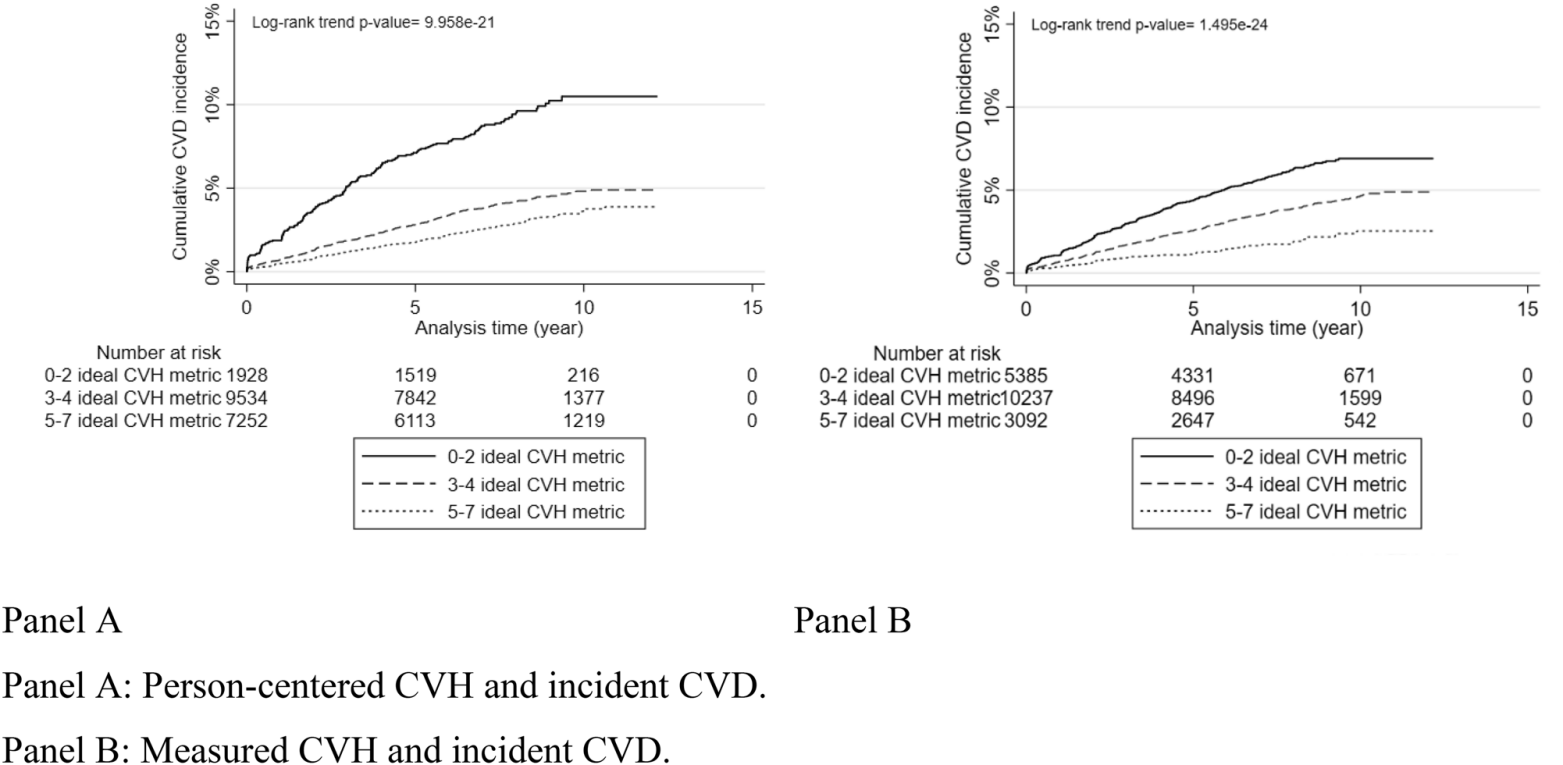
Kaplan–Meier curves showing CVD incidence for person-centered and measured CVH. Panel A: Person-centered CVH and incident CVD. Panel B: Measured CVH and incident CVD.

**Figure 3:**
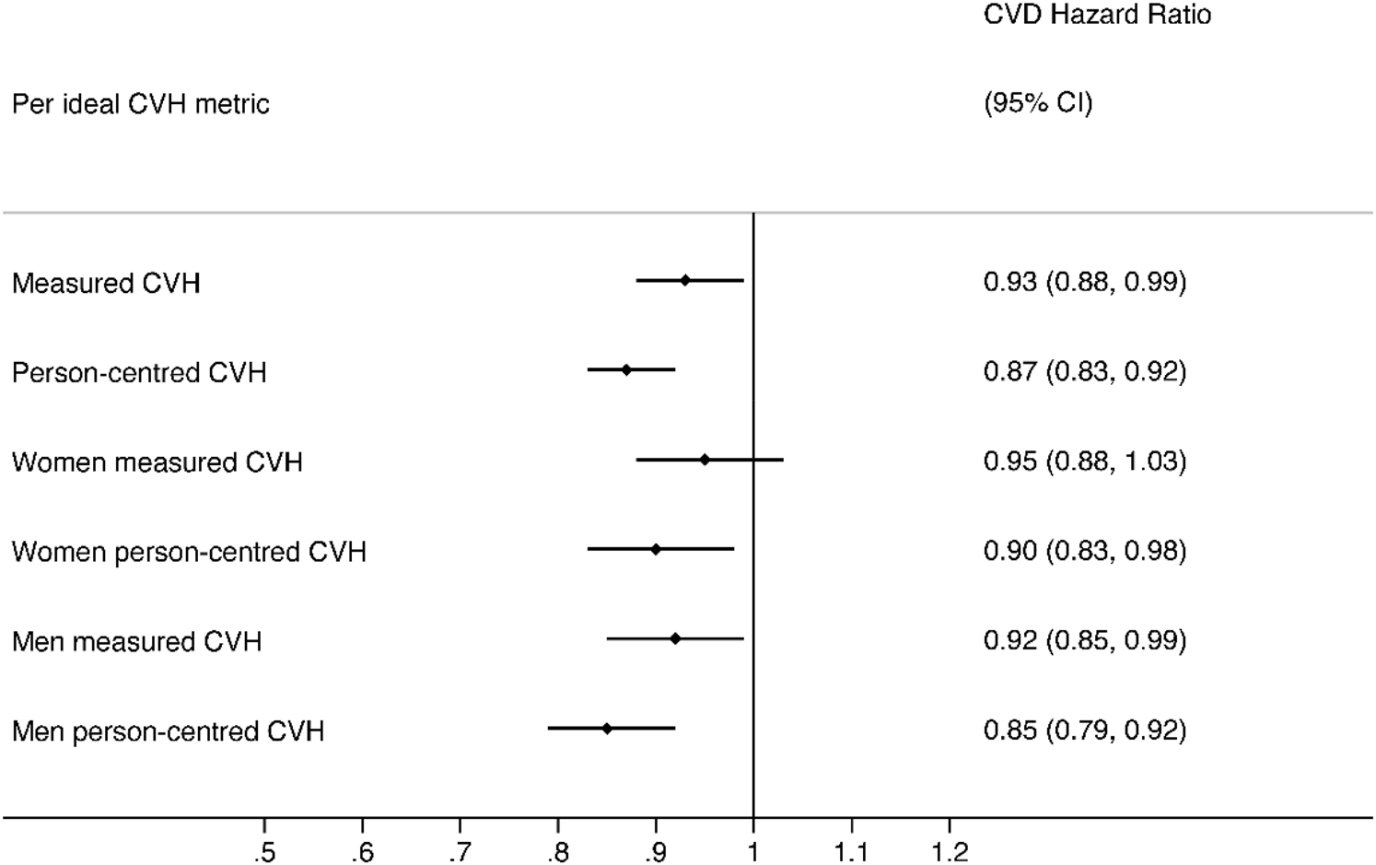
Hazard ratios and 95% confidence intervals for incident CVD per unit increase in ideal measured and person-centered CVH metrics, overall and by sex. Hazard ratios and 95% confidence intervals were estimated by Cox proportional models using age as a timescale, stratified by birth year (5-year intervals), and adjusted for baseline sex (when appropriate), cohabitation status, educational attainment, occupational category, alcohol use, and CVD family history.

After a median follow-up time of 8.05 (25th;75th percentiles: 6.19; 9.30) years, 749 incident CVD events occurred, including 724 non-fatal CVD events, comprising 351 coronary heart disease CHD and 373 stroke events, yielding an incidence rate of 5.31 per 1000 person-years, 95% CI [4.92; 5.72]. Kaplan- Meier curves suggest that the CVD incidence rate decreased progressively from low to high person-centered CVH (Fig. 2. Panel A, p for log-rank trend < 0.05), and measured CVH (Fig. 2. Panel B, p for log-rank trend < 0.05) levels. In multivariable models, there was a 7% (HR = 0.93, 95% CI [0.88; 0.99]) and 13% (HR = 0.87, 95% CI [0.83; 0.92]) risk reductions of CVD per additional increase in measured and person-centered ideal LS7 metrics, respectively (Table 5). Also, there was a 29% (HR = 0.71, 95% CI [0.53; 0.95]) and a 40% (HR = 0.60, 95% CI [0.49; 0.74]) risk reduction of CVD events for high (5 ideal metrics or more) versus low (2 ideal metrics or less) category of measured and person-centered CVH, respectively (Table 5). The findings suggest no sex modification effect between person-centered or measured CVH and incident CVD (Fig. 3), and no educational attainment level (p for interaction = 0.60 and 0.20) or occupational category moderating effect (p for interaction = 0.85 and 0.60), not shown.

**Table 5:** Hazard ratios of person-centered and measured Life’s Simple 7 cardiovascular health for incident cardiovascular disease (CVD).

	Person-centered CVH		Measured CVH	
	CVD incidence rate per 1000 person-years	HR 95% CI	CVD incidence rate per 1000 person-years	HR 95% CI
	95% CI		95% CI	
LS7 CVH levels				
Low	12.32 [10.57; 14.28]	1 (ref.)	6.33 [5.65; 7.06]	1 (ref.)
Intermediate	5.21 [4.70; 5.76]	0.71 [0.59; 0.85]	4.06 [3.67; 4.48]	0.94 [0.81; 1.10]
High	3.68 [3.20; 4.20]	0.60 [0.49; 0.74]	2.07 [1.59; 2.65]	0.71 [0.53; 0.95]
Per number of ideal LS7 metrics (0–7)				
Total	NA	0.87 [0.83; 0.92]	NA	0.93 [0.88; 0.99]
Women	NA	0.90 [0.83; 0.98]	NA	0.95 [0.88; 1.03]
Men	NA	0.85 [0.79; 0.92]	NA	0.92 [0.85; 0.99]
Sex interaction p-value		0.3		0.33

*LS7* life’s simple 7, *CVH* cardiovascular health, *HR* hazard ratio, *CVD* cardiovascular disease, *NA* not applicable.

Hazard ratios and 95% confidence intervals were estimated by Cox proportional models using age as a timescale, stratified by birth year (5-year intervals), adjusted for baseline sex, cohabitation status, educational attainment, occupational category, alcohol use, and CVD family history.

Levels of measured/person-centered LS7 CVH: low (0–2 ideal metrics), intermediate (3–4 ideal metrics), or high (5–7 ideal metrics).

### Additional analyses results

The results of additional analyses are reported in Supplementary Table S2. The estimates remained consistent with those reported in the main analysis after excluding first-year incident CVD (n = 139 events), considering the number of ideal biological metrics only (range 0–3), considering the complete case analysis, or accounting for competing risk by non-CVD mortality. Also, measured and person-centered CVH were equally and inversely related to non-CVD mortality (n = 169 deaths). Moreover, the estimated population attributable preventive fraction (PPF) is 29.37%, 95% CI [7.04; 46.33] and 15.54%, 95% CI [5.52; 24.49] for the measured and person-centered CVH, respectively.

The change analysis of person-centered CVH included 126,871 participants evaluated at baseline (2009 to 2014) and after 5 years, excluding CVD events in the interval. Overall, 56.04% maintained their CVH level at the 2 time points, including 3.54% at low CVH (0–2 ideal LS7 metrics), 59.29% at intermediate CVH (3–4 ideal LS7 metrics), and 37.17% at high CVH (5–7 ideal LS7 metrics). Secondly, 25.61% declined in CVH, including 67.90% and 25.39% of participants initially with intermediate and high CVH. Thirdly, 18.36% improved their CVH, including 20.40% and 42.89% of participants initially with low and intermediate CVH. Over a median follow-up of 6.98 years after the second CVH evaluation, 955 CVD events occurred. The risk of CVD decreased by 13% per increase in ideal person-centered LS7 metric independently of the baseline number of ideal person-centered LS7 metrics (Supplementary Table S3).

## Discussion

In this prospective e-cohort study conducted in 18,714 adult CVD-free adults, we report for the first time the results of a head-to-head comparison of person-centered CVH vs. measured CVH regarding their concordance and their respective associations with incident CVD in the same sample. The findings suggest that person-centered CVH data is a reliable approximate of measured CVH, which may have important implications for the spread of primordial prevention in the community.

Several points indicate that person-centered CVH is a reliable approximate of measured CVH. Firstly, concordance between person-centered and measured CVH was high, both for the global CVH and for most (biological) metrics. Still, person-centered data on ideal cholesterol status (i.e. non-hypercholesterolemic) exhibited the least concordance with its measured counterpart (i.e. ideal total serum cholesterol). Insufficient surveillance and public awareness of hypercholesterolemia in middle-aged adults may contribute to the lower concordance of the person-centered and measured hypercholesterolemia compared to hypertension and T2D^21,22^. So far, only one previous cross-sectional study conducted in 8,943 middle-aged Brazilians has compared person-centered and measured CVH^23^. In that study, the sensitivity and specificity of the person-centered CVH were 92.4 and 48.5%, compared to 93.6 and 72.1% in the current study. Direct comparisons between the two studies are difficult owing to the differences in high CVH level definition, ≥ 4 ideal metrics vs. ≥ 5 ideal metrics in the current study. We extended the previous study results to twice the number of participants, by using weighted concordance analysis, allocating more weight to misclassifications in higher CVH categories, and conducting a concordance analysis for each biological CVH metric.

Second, as for the measured CVH, the person-centered CVH was associated with a risk reduction of CVD per additional increase in ideal LS7 metric. Of note, however, the risk reduction per additional ideal metric was overestimated compared to the measured CVH (13% vs. 7%). Such difference is likely due to misclassification of person-centered biological LS7 metrics, since behavioral LS7 metrics are common to both person-centered and measured CVH. This is illustrated in the supplementary analyses, comparing the associations between person-centered and measured biological CVH with incident CVD. The findings on person-centered CVH and incident CVD are nevertheless consistent with those reported in postmenopausal women, even though no comparison with measured CVH was conducted in that study^24^.

Lastly, change in person-centered CVH (i.e. increase in the number of ideal metric) is associated with CVD risk reduction independent from baseline CVH, in accordance to what has been previously found for the change in measured CVH in other studies^25^.

The findings may have global health implications for primordial prevention. The results of the concordance analysis and the head-to-head comparison of the associations with CVD events suggest that person-centered CVH may represent an acceptable alternative to measured CVH. A person-centered CVH method has advantages over measured CVH defined by clinical examination and laboratory-based biomarkers: it is economical and more feasible to monitor, both on the individual and mass levels. Additionally, person-centered CVH may facilitate self-evaluation of CVH in the population, which may help incentivize the participation of individuals in their CVD risk assessment and prevention, and empower their decision to adopt healthier behaviors, i.e. participatory medicine^26^. In this context, the advent of wearable devices that measure physical activity, heart rate, or sleep patterns represents opportunities to self-evaluate CVH and endorse active person-centered CVH monitoring. From the health care provider’s point of view, a person-centered CVH may facilitate closer monitoring of CVH in the population, and screening of vulnerable sub-populations, and it may also help launch appropriate interventions to target CVH population prevention goals. For instance, in the secondary prevention setting, e-health interventions have been proposed to improve chronic disease outcomes^27,28^. Also, self-monitoring and telemonitoring have been suggested to be cost-effective management approaches for BP reduction in the UK^29^. Similar approaches might be extended to primordial and primary CVD prevention. Furthermore, person-centered CVH might be particularly useful in low and middle-resource settings or medically underserved high-income regions, where access to preventive cardiovascular services is challenging. However, future studies should evaluate the concordance of person-centered vs. measured CVH in LMICs or undeserved high-income areas finally, we estimated that 15.54% of CVD events could be prevented under the hypothesis that all of the population would acquire an ideal person-centered CVH. However, these results represent a conservative estimate, approximately half of that derived from the measured CVH in the current study population and the US adult population^30^.

## Limitations

The high concordance between person-centered and measured CVH may be in part due to the inclusion of more participants who are women and of higher socioeconomic level compared to the general French population. Therefore, study participants may have been more aware of their hypertensive or diabetic statuses than the general population. However, it is unlikely to have impacted the comparative associations between person- centered or measured CVH and CVD events, since direct comparison of person-centered and measured CVH was conducted for the same individuals. The over-representation of women in this study may also have contributed to not detecting sex interactions in the associations between person-centered or measured CVH and CVD, despite prior evidence in sex-difference in the CVH distribution^31^. Measured biological LS7 metrics were assessed once, precluding the estimation of the concordance of the measured vs. person-centered CVH change and their respective associations with incident CVD. Despite model adjustment for potential confounders, residual confounding cannot be ruled out when comparing the association of measured and person-centered CVH with incident CVD. At the time of the study, the participants’ ethnicity data were not available. The present study was conducted in a high-resource setting, future studies should examine the relevance of person-centered CVH in different global health contexts.

## Conclusions

Person-centered CVH represents a reliable alternative to measured CVH and therefore might be useful for monitoring population CVH, a global health priority, particularly in underserved HIC areas and LMICs.

## Supplementary Information

Supplementary Information.

## Data Availability

Researchers from public institutions can submit a collaboration request to Dr. Mathilde Touvier via collaboration@etude-nutrinet-sante.fr, including information on the institution and a brief description of the project.All requests will be reviewed by the steering committee of the NutriNet-Santé study. If the collaboration is accepted, a data access agreement will be necessary, and appropriate authorizations from competent administrative authorities may be needed. In accordance with existing regulations, no personal data will be accessible. The analyses code can be requested from the authors.

## Abbreviations

CVH: Cardiovascular health
LS7: Life’s simple 7
LMICs: Low and middle-income countries
BMI: Body mass index
T2D: Type 2 diabetes mellitus
CVD: Cardiovascular disease
SNIIRAM: The medico-administrative databases of the national health insurance (France)
BP: Blood pressure
PPV: Positive predictive value
NPV: Negative predictive value
HR: Hazard ratio
CI: Confidence interval
