# Supplementary material for: Person-centered and measured life’s simple 7 cardiovascular health concordance and association with incident cardiovascular disease
Source: Sci Rep. 2023 Mar 31;13:5247. doi: 10.1038/s41598-023-32219-x (PMC10066211; doi:10.1038/s41598-023-32219-x)
Supplement: Supplementary file 1 — Supplementary Information. [file 41598_2023_32219_MOESM1_ESM.docx]

Supplementary material

Supplementary Figure S1. Study timeline and main dates.
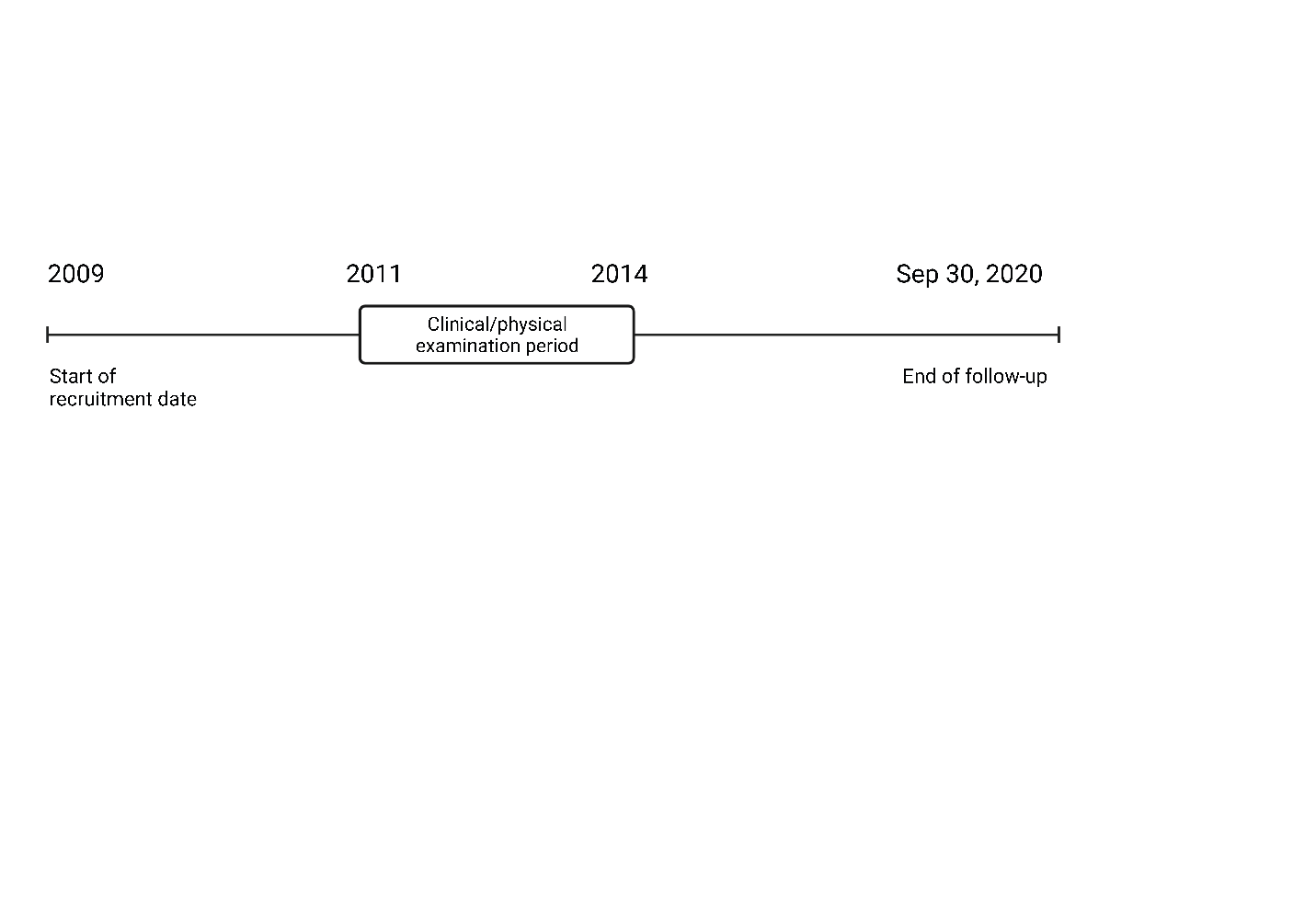


Supplementary Table S1. Distribution of person-centered and measured Life’s Simple 7 (LS7 ) ideal cardiovascular health (CVH) metrics.

|  | **Person-centered CVH** | **Measured CVH** |
| --- | --- | --- |
| **Ideal non-smoking, n (%)** | 8637 (46.15%) | |
| **Ideal BMI, n (%)** | 11406 (60.95%) | |
| **Ideal physical activity, n (%)** | 6665 (35.62%) | |
| **Ideal diet, n (%)** | 70 (0.37%) | |
| **Ideal blood glycemia, n (%)** | 16150  (86.30%) | 18299  (97.78%) |
| **Ideal blood pressure, n (%)** | 11399  (60.91%) | 15048  (80.41%) |
| **Ideal blood cholesterol, n (%)** | 16587  (88.63%) | 15624  (83.49%) |

Being self-reported, behavioral metrics were common to person-centered and measured CVH. Ideal levels of behavioural metrics corresponded to: Never or former smoker ≥ 12 months, BMI < 25 kg/m2, physical activity: MET≥ 75 min/week of vigorous activity, MET ≥ 150 min/week of moderate activity, or a combination of the two, Ideal diet: ≥ 4 optimal diet items out of the following 5 items, the equivalent of 5 servings per day of fruits and vegetables or 400 gm/day, ≥ 2 times/week fish consumption or average consumption of ≥ 100 gm/day, average sodium consumption < 1.5 gm/day, average fiber intake ≥ 25 gm/day, average intake of sugary drinks ≤ 152 ml/day. Ideal levels of biological metrics corresponded to: absence of diagnoses, medication, or treatment for for person-centered factors. For measured health factors: untreated < 100 mg/dL was ideal measured blood glucose, untreated SBP < 120 and DBP < 80 mmHg for measured blood pressure, and untreated < 200 mg/dL for measured total blood cholesterol.

|  | Measured CVH  HR [95% CI] | Person-centered CVH  HR [95% CI] |
| --- | --- | --- |
| Excluding first CVD events occurring in the 1^st^ year of follow-up | 0.93 [0.87; 0.99] | 0.88 [0.83; 0.94] |
| Biological CVH metrics (0-3) | 0.91 [0.83; 0.99] | 0.85 [0.81; 0.89] |
| Excluding missing CVH metrics: complete case analysis | 0.93 [0.88; 0.99] | 0.87 [0.82; 0.92] |
| Non-CVD mortality | 0.88 [0.78; 0.99] | 0.88 [0.78; 0.98] |
| Competing risk analysis | 0.92 [0.87; 0.98] | 0.86 [0.82; 0.91] |

Supplementary Table S2. Additional analyses.

Hazard ratios, 95% confidence intervals estimated by multivariable Cox models using age as a timescale, stratified by birth year (5-year intervals), adjusted for baseline sex, cohabitation status, educational attainment, occupational category, alcohol use, and CVD family history.

Measured ideal biological metrics are untreated fasting plasma glucose < 100 mg/dL, untreated systolic and diastolic blood pressure < 120/80 mmHg, and untreated total serum cholesterol < 200 mg/dL. Person-centered ideal metrics are the absence of medication, treatment, or diagnosis for type 2 diabetes, blood pressure, and hypercholesterolemia.

Fine and Gray multivariable model sub-distribution hazard ratios for incident CVD and their 95% confidence interval, using non-CVD mortality as a competitive outcome.

Supplementary Table S3. 5-year change in person-centered Life‘s Simple 7 (LS7) cardiovascular health (CVH) and incident cardiovascular disease (CVD).

| **n= 126,871, CVD events, n= 955** | **Hazard ratio 95% CI** |
| --- | --- |
| **Per change in ideal person-centered LS7 CVH metric over 5 years** | 0.87 [0.82; 0.92] |
| **Per ideal person-centered LS7 CVH metric at baseline** | 0.88 [0.83; 0.93] |

LS7: Life’s Simple 7. CVH: cardiovascular health. HR: Hazard ratio. CVD: cardiovascular disease. Hazard ratios and 95% confidence intervals were estimated by Cox proportional models using age as a timescale, stratified by birth year (5-year intervals), adjusted for baseline sex, cohabitation status, educational attainment, occupational category, alcohol use, and CVD family history. Person-centered CVH change was measured 5 years after baseline. The model for CVH change was adjusted for baseline person-centered CVH.
